# Longitudinal associations between Big Five personality and insomnia: evidence based on a 4-year perspective cohort study among community residents

**DOI:** 10.3389/fpsyg.2025.1569036

**Published:** 2025-05-09

**Authors:** Ziming Shao, Zhen Wei, Meiqi Wang, Yifan Chen, Yazhuo Qi, Zihan Zhou, Yunxi Zhong, Afei Qin, Yingyue Xu, Kaixian Wang, Wenyu Wang, Long Sun

**Affiliations:** ^1^Centre for Health Management and Policy Research, School of Public Health, Cheeloo College of Medicine, Shandong University, Jinan, China; ^2^National Health Commission of China (NHC) Key Laboratory of Health Economics and Policy Research (Shandong University), Jinan, China

**Keywords:** longitudinal study, personality, insomnia, community residents, cross-lagged relations

## Abstract

**Introduction:**

While the association between personality traits and insomnia has been confirmed, the longitudinal relationship between them remains unclear.

**Methods:**

This is a 4-year longitudinal cohort study conducted among rural community residents (*N* = 482) in Shandong Province, China.

**Results:**

This study found that neuroticism (*β* = 0.175, *p* < 0.001), extraversion (*β* = −0.146, *p* < 0.001), and conscientiousness (*β* = −0.168, *p* < 0.001) at baseline had statistically significant longitudinal associations with insomnia at follow-up. In addition, insomnia at baseline had statistically significant longitudinal correlations with neuroticism (*β* = 0.142, *p* < 0.01), extraversion (*β* = −0.209, *p* < 0.001), and agreeableness (*β* = −0.122, *p* < 0.01) at follow-up.

**Discussion:**

This study showed that both neuroticism and extraversion had a bidirectional relationship with insomnia. Insomnia had a one - way longitudinal correlation with agreeableness, and conscientiousness had a one - way longitudinal correlation with insomnia. The longitudinal correlation between openness and insomnia was not supported in this study.

## Introduction

Personality refers to the sum of psychological characteristics, that distinguish an individual from others (Mucci et al., 2014). It is relatively stable, and influences a person’s behavior patterns (Yokoyama et al., 2021). In recent decades, many studies have proved that individuals’ personality traits are associated with a range of health problems, such as cardiovascular disease, diabetes, hypertension, and metabolic syndrome (Kok et al., 2016; Wang et al., 2020; Laaboub et al., 2022; Akram et al., 2023; Zamani Sani et al., 2023; Yan and Li, 2024). Insomnia, a common health problem, has also been found to be associated with personality traits (Dekker et al., 2017; Zhang N. et al., 2023). However, when we reviewed the previous studies about the associations between insomnia and personality traits, the longitudinal relationships between them cannot be defined.

The reasons why the longitudinal relationships between insomnia and personality traits cannot be confirmed are complex. The first reason was that most of the studies were designed with a cross-sectional design. This type of study design alone does not allow us to draw conclusions about longitudinal relationships. For example, a study conducted in the United States found that insomnia was associated with higher neuroticism (Emert and Lichstein, 2017), a study conducted in Italy found that lower conscientiousness and higher neuroticism were associated with insomnia (Fabbri et al., 2022), and a study conducted in Netherlands found that neuroticism was positively correlated with insomnia and openness was negatively correlated with insomnia (Dekker et al., 2017). These studies were all limited by their cross-sectional design and were unable to explore the longitudinal relationship between insomnia and personality.

Another reason is that, despite some studies having utilized a longitudinal cohort design, their representation was constrained due to the fact that the majority of these studies were conducted within special populations. For example, two longitudinal studies conducted among nurses in Norway showed that personality was a significant predictor of insomnia (Vedaa et al., 2016; Sørengaard et al., 2019), and a study indicating that neuroticism was a strong predictor of insomnia among police employees in Norway (Sørengaard et al., 2022).

Thirdly, due to the relative stability of personality, it was difficult to observe the effect of insomnia on personality traits in short-term follow-up studies. For example, a study conducted in the United States with follow-up intervals of 6 months showed that no significant changes in personality could be observed with short-term follow-up (Luong et al., 2015), and the study in the United States, with a follow-up interval of 2.5 years, also showed that no significant changes in personality could be observed with short-term follow-up (Cooper et al., 2014).

Fourthly, considering the multiple dimensions of personality and the different relationships between personality traits and insomnia, inappropriate personality classification methods could confuse the relationship between personality and insomnia. For example, a study in Poland used the Type D personality, which focuses relatively exclusively on social inhibition and negative affect and may have overlooked other personality factors that may influence insomnia (Domagalska et al., 2021).

To fill the gaps, a 4-year longitudinal cohort study conducted among rural community residents (*N* = 482) to investigate the longitudinal associations between insomnia and personality traits. By doing so, the study aims to furnish evidence supporting the longitudinal relationships between insomnia and personality, thereby offering a fresh vantage groundwork for further exploration in the realm of personality and insomnia research.

## Methods

### Study participants and data collection

This is a prospective cohort study conducted by face-to-face household interviews in rural areas of Shandong Province, China. In November 2019, the baseline study was initiated with 879 rural residents participating. Subsequently, the follow-up study was carried out in August 2023, with 489 rural residents taking part. After accounting for various factors such as missing data on personality or insomnia (7 participants), deaths (51participants), migration to town (145participants), and loss to follow-up (194 participants), a total of 482 participants were included in the current study.

The researchers obtained support from local village doctors in the township health centers before commencing the survey. During the survey, rural residents willingly participated and provided written informed consent. For illiterate participants, their legal caregivers assisted in completing the consent form. Data collection was conducted through face-to-face interviews by trained students who were well-versed in the study objectives and questionnaire content. Following the interviews, all questionnaires were reviewed, and those with missing data were revisited and completed.

### Measures

#### Personality

Personality psychologists have concluded that five main dimensions explain most of the individual differences in personality traits (McCRAE and Costa, 1991). Neuroticism Extraversion Openness Personality Inventory (NEO-PI) developed by Costa and McCrae (1985 in Schipper et al., 2010) is a widely used big five personality measurement tool. In 1989, they published a simplified version of NEO-PI with only 60 items, named Neuroticism Extraversion Openness Personality Five-Factor Inventory (NEO-FFI). Costa and McCrae (1992 in Aluja et al., 2005) updated the 240-item version of the Personality Scale to the NEO-PI-R and introduced a simplified version (also named NEO-FFI). The NEO-FFI was developed by culling the items from the NEO-PI-R that demonstrated the best discriminant and convergent validity with the NEO-PI-R valid max factors (Scandell and Wlazelek, 1999). In this study, we used the upgraded NEO-FFI, a concise 60-item version of the Big Five personality traits encompassing five dimensions: Neuroticism, Extraversion, Openness, Agreeableness, and Conscientiousness (Qin et al., 2024). The questionnaire comprises 60 questions that are rated on a 5-point Likert scale, spanning from strongly agree to strongly disagree. The responses to the NEO-FFI items are scored from 1 to 5, with each score reflecting the individual item and its corresponding dimension (Choi et al., 2019). For more detailed information regarding each specific item and its corresponding answer value, please refer to the NEO-FFI manual authored by Costa and McCrae (1992). The reliability and validity of this scale have been well validated in the adult population in China (Yao and Liang, 2010).

#### Insomnia

The Athens Insomnia Scale (AIS) is an 8-item self-report questionnaire used to assess the severity of insomnia. It covers various aspects of sleep difficulties experienced in the past month, including sleep initiation, nocturnal awakenings, early morning awakening, total sleep duration, sleep quality, well-being, overall functioning, and daytime sleepiness (Fan et al., 2024). The Chinese version of AIS has demonstrated high internal consistency, reliability and validity, and is widely used (Sun et al., 2011). Scores on the AIS range from 0 to 24, with each item scored on a scale of 0 to 3 (0 = none, 1 = lowest, 2 = significant, and 3 = severe). A total score of 6 points or higher is considered critical for diagnosing insomnia with clinical significance (Zhang X. et al., 2023). In this scale, a higher score indicates more severe insomnia.

#### Covariates

Covariates mainly included sociodemographic variables and behavioral lifestyle variables at baseline. Sociodemographic variables included gender, age, body mass index (BMI) groups, marital status, solitude status, education, and current employment status. Behavioral lifestyle variables included tea consumption, alcohol consumption, smoking status, and frequency of physical activity. Gender was measured as male (0) and female (1). Marital status included unmarried/separated/widowed (0) and married (1). Current employment status was categorized as “no” (0) and “yes” (1), where “yes” indicates having a job, and solitude status also included “no” (0) and “yes” (1), where “yes” indicates living alone. Education was classified into three categories: illiteracy, primary school, and middle school and above. BMI was calculated by height (m) and weight (kg). Due to the sample composition, BMI was divided into four groups: normal weight (BMI < 18.5 kg/m^2^), underweight (18.5 kg/m^2^ ≤ BMI < 24 kg/m^2^), overweight (24 kg/m^2^ ≤ BMI < 28 kg/m^2^), and obese (BMI ≥ 28 kg/m^2^) (Lee et al., 2018). Drinking tea was classified as “no” (0) and “yes” (1). Former or current smoking and drinking both contained “no” (0) and “yes” (1). Frequent physical activity was divided into “no” (0) and “yes” (1) which was judged by whether it is outdoors three or more times a week.

### Statistical analysis

SPSS (version 27.0) and Mplus (version 8.3) were used for data analysis. Descriptive statistics, such as means and standard deviations (SD) for continuous variables, and numbers and percentages for categorical variables, were utilized for summarization. Descriptive statistics were performed on the sample to explore the relationship between five personality traits and insomnia by calculating correlation coefficients among key study variables. Cross-lagged models were constructed to further investigate the relationship between the five personality traits and insomnia. Covariates such as age, gender, and BMI were included in the model. Additionally, a sensitivity analysis was conducted to examine the trends between the five personality traits at baseline and the AIS at follow-up using linear regression. All tests were two-tailed, and statistical significance was set at a *p*-value of less than 0.05.

## Results

### Descriptive statistics and single analysis

In this study, there were 482 participants from rural areas in Shandong Province, China. Table 1 presents the sample characteristics and single-factor analyses. The majority were elderly, with a mean age of 63.54 (SD = 13.25), of which 63.3% were female. Approximately 85.7% of the participants were married, while 14.3% were categorized as others (single, divorced, and widowed). The educational level of participants was generally low of which 39.4% were Illiteracy, 31.1% were primary and 29.5% were middle and above. The majority were employed and living with others, accounting for 82.2 and 88.4%, respectively. According to the BMI-related categories, the proportions classified as underweight, normal, overweight, and obese were 3.1, 52.9, 32.8, and 12.2%, respectively. There were 25.3% drinking tea, 18.5% drinking or past drinking, and 24.1% smoking or past smoking. 51.5% of the people there had frequent outdoor activities. As shown in Table 1, age (*r* = 0.29, *p* < 0.001), marital status (*F* = 16.31, *p* < 0.001), education (*F* = 23.58, *p* < 0.001).

**Table 1:** Sample characteristics and single analysis for the factors associated between personality traits and insomnia at follow-up.

Variables	Mean ± SD/n (%)	NE at follow-up		EX at follow-up		OP at follow-up		AG at follow-up		CO at follow-up		AIS at follow-up	
		Mean ± SD	*t*/*F*/*r*	Mean ± SD	*t*/*F*/*r*	Mean ± SD	*t*/*F*/*r*	Mean ± SD	*t*/*F*/*r*	Mean ± SD	*t*/*F*/*r*	Mean ± SD	*t*/*F*/*r*
*N*	482 (100)												
Age	63.54 ± 13.25		0.53		−0.57		0.54		−0.01		−1.56***		0.29***
Gender			13.63***		5.35*		1.37		1.37		7.56**		3.74
Male	177 (36.70)	25.25 ± 7.47		43.92 ± 6.23		35.95 ± 4.44		43.96 ± 4.27		47.36 ± 6.01		3.49 ± 5.13	
Female	305 (63.30)	28.15 ± 8.75		42.37 ± 7.58		35.46 ± 4.34		43.50 ± 4.07		45.77 ± 6.20		4.55 ± 6.17	
Married status			6.26*		3.66		0.54		0.33		4.87*		16.31***
Married	413 (85.70)	26.70 ± 8.08		43.19 ± 7.10		35.58 ± 4.36		43.71 ± 4.13		46.61 ± 6.17		3.73 ± 5.60	
Others	69 (14.30)	29.42 ± 9.91		41.42 ± 7.28		36.00 ± 4.49		43.41 ± 4.27		44.84 ± 6.01		6.73 ± 6.49	
Educational attainments			5.00**		1.57		0.00		1.77		1.38		23.58***
Illiteracy	190 (39.40)	28.53 ± 8.90		42.24 ± 7.29		35.63 ± 4.58		43.65 ± 4.11		46.11 ± 6.12		6.24 ± 7.03	
Primary	150 (31.10)	26.50 ± 8.04		43.26 ± 7.23		35.66 ± 4.25		43.24 ± 4.31		45.99 ± 5.74		3.37 ± 4.61	
Middle and above	142 (29.50)	25.77 ± 7.87		43.54 ± 6.82		35.63 ± 4.27		44.15 ± 3.99		47.07 ± 6.65		2.20 ± 4.11	
Occupation			2.77		2.88		0.95		0.01		2.98		3.06
Yes	396 (82.20)	26.79 ± 8.11		43.19 ± 7.09		35.55 ± 4.41		43.66 ± 4.16		46.58 ± 6.11		3.94 ± 5.63	
No	86 (17.80)	28.45 ± 9.60		41.76 ± 7.30		36.06 ± 4.24		45.31 ± 6.38		45.31 ± 6.38		5.15 ± 6.61	
Living alone			2.33		4.27*		2.15		0.07		7.58**		3.75
Yes	56 (11.60)	28.70 ± 8.49		41.09 ± 6.86		36.45 ± 4.13		43.54 ± 3.93		44.23 ± 4.94		5.57 ± 6.04	
No	426 (88.40)	26.88 ± 8.39		43.18 ± 7.15		35.53 ± 4.40		46.63 ± 6.27		46.63 ± 6.27		3.97 ± 5.78	
BMI			2.97*		0.30		0.30		0.21		4.70**		1.39
Underweight (<=18.4)	15 (3.10)	32.07 ± 8.70		42.73 ± 5.50		34.60 ± 3.36		43.73 ± 3.47		43.40 ± 7.02		3.67 ± 4.48	
Normal (18.5–23.9)	250 (52.90)	28.86 ± 8.47		43.07 ± 7.11		35.64 ± 4.52		43.67 ± 4.38		46.74 ± 5.81		4.42 ± 6.28	
Overweight (24.0–27.9)	158 (32.80)	26.36 ± 8.28		43.06 ± 7.24		35.70 ± 4.32		43.81 ± 3.84		46.88 ± 6.15		3.46 ± 4.98	
Obese (≥28.0)	59 (12.20)	28.71 ± 8.04		42.12 ± 7.50		35.75 ± 4.25		43.31 ± 4.17		44.05 ± 6.93		5.02 ± 6.15	
Drinking tea			4.93*		2.83		0.05		3.35		2.61		2.30
Yes	122 (25.30)	25.63 ± 0.72		43.88 ± 0.61		35.71 ± 0.41		44.26 ± 0.39		47.13 ± 0.55		3.47 ± 0.48	
No	360 (74.70)	27.58 ± 0.45		42.62 ± 0.38		35.62 ± 0.23		43.47 ± 0.21		46.09 ± 0.33		4.39 ± 0.32	
Former/Current drinking alcohol			5.01*		4.15*		1.50		0.56		1.81		1.23
Yes	89 (18.50)	25.29 ± 7.13		44.33 ± 6.09		36.15 ± 4.29		43.97 ± 4.26		47.15 ± 5.90		3.54 ± 4.78	
No	393 (81.50)	27.49 ± 8.63		42.62 ± 7.33		35.52 ± 4.40		43.60 ± 4.12		46.17 ± 6.23		4.30 ± 6.04	
Former/Current smoker			4.04*		2.24		0.78		0.14		3.70		0.25
Yes	116 (24.10)	25.72 ± 7.57		43.80 ± 5.79		35.95 ± 4.19		43.54 ± 4.07		47.31 ± 5.34		3.92 ± 5.32	
No	366 (75.90)	27.52 ± 8.63		42.66 ± 7.51		35.54 ± 4.44		43.71 ± 4.18		46.05 ± 6.39		4.23 ± 5.98	
Frequent physical activity			4.75*		1.82		4.83*		0.44		1.56		0.14
Yes	248 (51.50)	27.90 ± 9.11		42.51 ± 7.55		35.22 ± 4.28		43.55 ± 3.92		46.01 ± 6.49		4.25 ± 5.92	
No	234 (48.50)	26.23 ± 7.52		43.39 ± 6.68		36.09 ± 4.45		43.80 ± 4.38		46.71 ± 5.81		4.06 ± 5.74	

***p < 0.001, **p < 0.01, *p < 0.05. NE stands for neuroticism. EX stands for extraversion. OP stands for openness. AG stands for agreeableness. CO stands for conscientiousness. AIS stands for Athens Insomnia Scale score.

### Correlation

Table 2 presents the results of the correlation analysis between the five personality traits and insomnia at baseline and follow-up, including the mean, standard deviation, and correlation of the key variables. The results reveal notable positive associations between the five personality traits and insomnia between two waves, except for the openness at follow-up with insomnia at baseline and the agreeableness at follow-up with insomnia at follow-up.

**Table 2:** Mean (M), standard deviation (SD), and bivariate correlations of study variables.

Variables	*M*	SD	1	2	3	4	5	6	7	8	9	10	11	t
1. NE at baseline	28.74	7.71												4.01***
2. NE at follow-up	27.09	8.41	0.37***											
3. EX at baseline	42.25	6.80	−0.70***	−0.28***										−1.88
4. EX at follow-up	42.94	7.14	−0.30***	−0.63***	0.34***									
5. OP at baseline	37.12	3.60	−0.25***	−0.05	0.30***	0.07								6.13***
6. OP at follow-up	35.64	4.38	−0.12**	−0.10*	0.14**	0.18***	0.12**							
7. AG at baseline	42.76	3.85	−0.43***	−0.12**	0.47***	0.13**	0.07	0.06						−3.63***
8. AG at follow-up	43.67	4.15	−0.05	−0.41***	0.07	0.28***	−0.05	−0.02	0.04					
9. CO at baseline	44.79	6.09	−0.57**	−0.18***	0.61***	0.19***	0.31***	0.03	0.55***	−0.04				−4.29***
10. CO at follow-up	46.35	6.17	−0.08	−0.46***	0.16***	0.49***	0.09*	0.12*	0.09	0.44***	0.15***			
11. AIS at baseline	3.35	4.93	0.34***	0.28***	−0.31***	−0.30***	−0.09*	−0.04	−0.10*	−0.13**	−0.18***	−0.13**		−3.13**
12. AIS at follow-up	4.16	5.83	0.32***	0.46***	−0.28***	−0.38***	−0.13**	−0.10*	−0.11*	−0.05	−0.28***	−0.10*	0.46***	

***p < 0.001, **p < 0.01, *p < 0.05. NE stands for neuroticism. EX stands for extraversion. OP stands for openness. AG stands for agreeableness. CO stands for conscientiousness. AIS stands for Athens Insomnia Scale score.

### Multiple linear analysis

**Figure 1:**
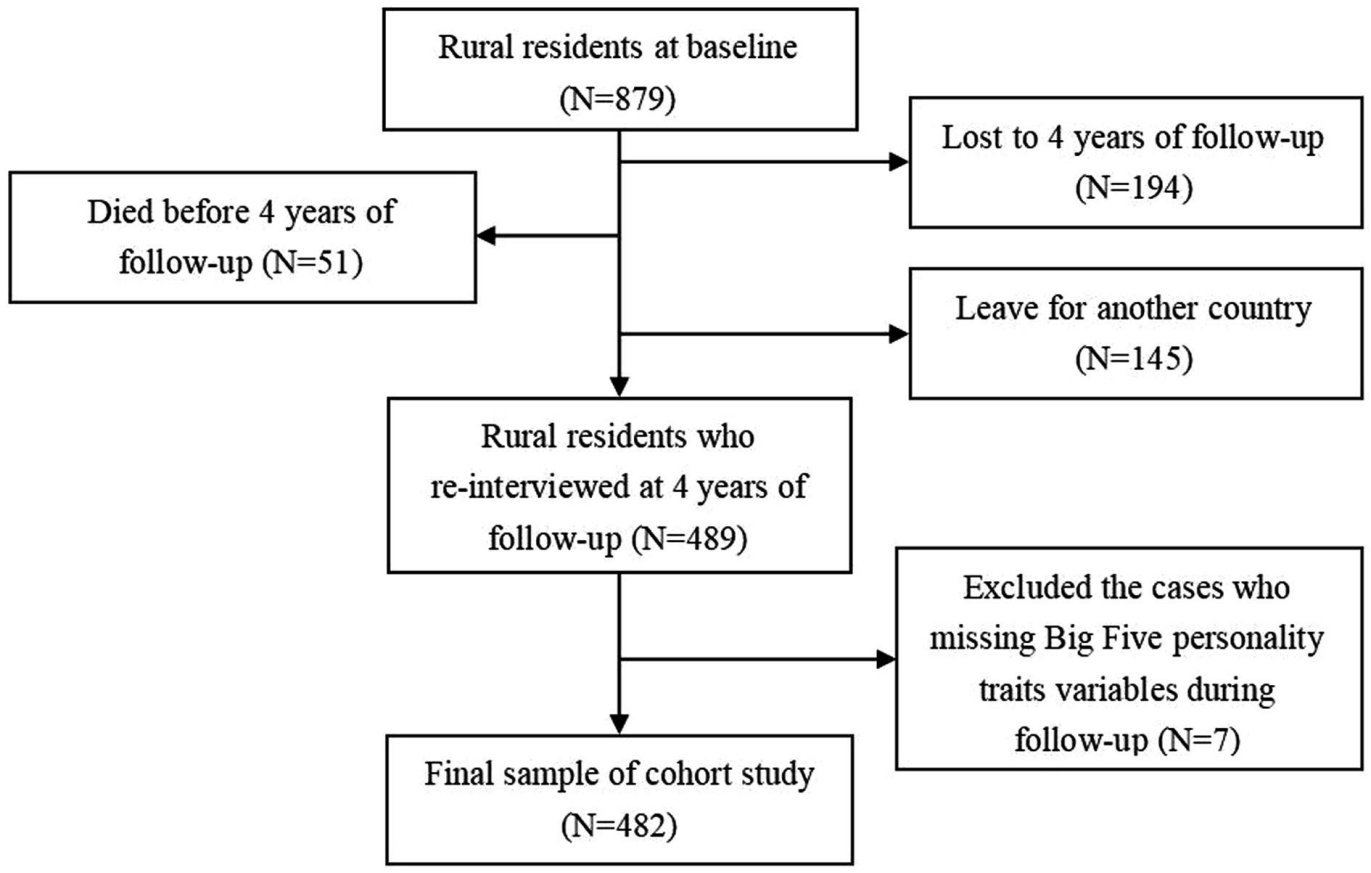
Flowchart of the procedure for data collection.

Multiple linear regression models were utilized to investigate the relationships between the five personality traits and insomnia, as summarized in Table 3. After adjusting for relevant social demographic and behavioral lifestyle variables, the results revealed positive associations between neuroticism at baseline and insomnia at follow-up (*β* = 0.12, *p* < 0.001), as well as negative associations between extraversion at baseline and insomnia at follow-up (*β* = −0.11, *p* < 0.01), and conscientiousness at baseline and insomnia at follow-up (*β* = −0.15, *p* < 0.001). However, when all five personality traits were placed in a single model, extraversion was not significant. Only neuroticism and conscientiousness remained significant. The multiple linear regression model, incorporating neuroticism (adjusted *R*^2^ = 0.32, *p* < 0.001), extraversion (adjusted *R*^2^ = 0.31, *p* < 0.001), and conscientiousness (adjusted *R*^2^ = 0.32, *p* < 0.001) as explanatory variable, displayed robust explanatory power for insomnia (Figure 1).

**Table 3:** Multiple linear regression analysis for the association between personality traits and insomnia.

Variables	AIS at follow-up (Model 1)	AIS at follow-up (Model 2)	AIS at follow-up (Model 3)	AIS at follow-up (Model 4)	AIS at follow-up (Model 5)
Age	0.08 (0.05,0.12)***	0.08 (0.04,0.12)***	0.08 (0.04,0.12)***	0.08 (0.04,0.12)***	0.07 (0.04,0.11)***
Gender	−0.37 (−1.54,0.80)	−0.55 (−1.72,0.64)	−0.46 (−1.64,0.73)	−0.45 (−1.63,0.74)	−0.37 (−1.54,0.80)
Marriage	−1.63 (−3.16,-0.11)*	−1.73 (−3.27,-0.20)*	−1.94 (−3.48,-0.40)*	−1.89 (−3.43,-0.34)*	−1.74 (−3.26,-0.22)*
Occupation	−0.80 (−1.95,0.36)	−0.80 (−1.96,0.36)	−0.93 (−2.10,0.23)	−0.94 (−2.10,0.23)	−0.77 (−1.92,0.38)
Live alone	−1.05 (−2.68,0.57)	−1.12 (−2.75,0.52)	−1.19 (−2.84,0.46)	−1.08 (−2.75,0.58)	−1.10 (−2.73,0.52)
Education (Ref. = Illiteracy)					
Primary school	−1.94 (−3.00,-0.89)***	−1.87 (−2.94,-0.81)***	−2.05 (−3.12,-0.98)***	−2.04 (−3.11,-0.97)***	−1.94 (−3.00,-0.88)***
Middle school and above	−2.68 (−3.76,-1.60)***	−2.72 (−3.80,-1.63)***	−2.73 (−3.82,-1.63)***	−2.77 (−3.86,-1.68)***	−2.66 (−3.74,-1.58)***
BMI (Ref. = Normal)					
Underweight	−1.17 (−3.72,1.38)	−1.33 (−3.90,1.23)	−1.38 (−3.97,1.21)	−1.28 (−3.87,1.31)	−1.33 (−3.88,1.22)
Overweight	−1.00 (−1.97,-0.02)*	−0.93 (−1.91,0.05)	−0.85 (−1.84,0.14)	−0.89 (−1.88,0.09)	−0.89 (−1.86,0.09)
Obese	0.65 (−0.75,2.05)	0.57 (−0.84,1.98)	0.62 (−0.80,2.05)	0.64 (−0.78,2.07)	0.63 (−0.78,2.03)
Drink tea frequently	−0.42 (−1.43,0.59)	−0.35 (−1.37,0.67)	−0.36 (−1.39,0.68)	−0.42 (−1.45,0.61)	−0.32 (−1.33,0.69)
Former/Current drinking alcohol	−0.04 (−1.47,1.40)	−0.08 (−1.53,1.36)	−0.17 (−1.63,1.28)	−0.24 (−1.69,1.22)	−0.09 (−1.52,1.35)
Former/Current smoker	−0.19 (−1.50,1.13)	−0.19 (−1.51,1.13)	−0.25 (−1.58,1.09)	−0.18 (−1.52,1.15)	−0.10 (−1.42,1.21)
Frequent physical activity	0.24 (−0.65,1.12)	0.26 (−0.63,1.16)	0.47 (−0.43,1.36)	0.40 (−0.50,1.30)	0.33 (−0.55,1.21)
NE	0.12 (0.06,0.18)***	—	—	—	—
EX	—	−0.11 (−0.18, −0.04)**	—	—	—
OP	—	—	−0.07 (−0.19, 0.06)	—	—
AG	—	—	—	−0.06 (−0.18, 0.05)	—
CO	—	—	—	—	−0.15 (−0.22, −0.07)***
AIS at baseline	0.37 (0.27, 0.47)***	0.39 (0.29, 0.49)***	0.43 (0.34, 0.53)***	0.43 (0.33, 0.53)***	0.41 (0.32, 0.51)***
Constant	−1.77 (−5.45, 1.92)	6.35 (2.11, 10.6)**	4.65 (−1.01, 10.3)	4.75 (−1.13, 10.63)	8.6 (4.01, 13.18)***
Adjusted *R*^2^	0.32***	0.31***	0.30***	0.30***	0.32***

***p < 0.001, **p < 0.01, *p < 0.05. NE stands for neuroticism. EX stands for extraversion. OP stands for openness. AG stands for agreeableness. CO stands for conscientiousness. AIS stands for Athens Insomnia Scale score.

### The cross-lagged model

**Figure 2:**
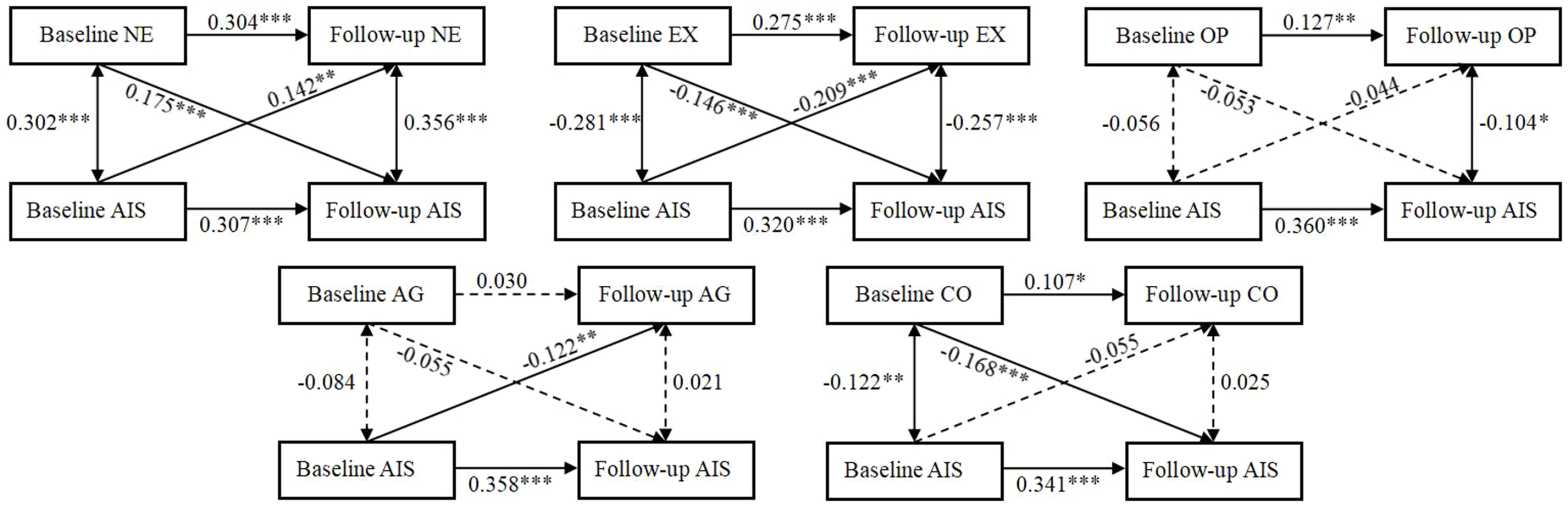
The standardized cross-lagged model between insomnia (AIS stands for Athens Insomnia Scale score) and five personality traits. NE stands for neuroticism, EX stands for extraversion, OP stands for openness, AG stands for agreeableness, and CO stands for conscientiousness; ***p < 0.001, **p < 0.01, *p < 0.05.

In order to investigate the longitudinal relationship between five personality traits and insomnia, five cross-lagged models were created incorporating variables like age, gender, BMI, and educational level. In the cross-lagged model of neuroticism and insomnia, the autoregressive paths of neuroticism (*β* = 0.304, *p* < 0.001) and insomnia (*β* = 0.307, *p* < 0.001) were all significant. In this model, the cross-lagged path from neuroticism at baseline to insomnia at follow-up (*β* = 0.175, *p* < 0.001) and the path from insomnia at baseline to neuroticism at follow-up (*β* = 0.142, *p* < 0.01) were significant. In the cross-lagged model of extraversion and insomnia, the autoregressive paths of neuroticism (*β* = 0.275, *p* < 0.001) and insomnia (*β* = 0.320, *p* < 0.001) were all significant. In this model, the cross-lagged path from extraversion at baseline to insomnia at follow-up (*β* = −0.146, *p* < 0.001) and the path from insomnia at baseline to extraversion at follow-up (*β* = −0.209, *p* < 0.001) were significant. In the cross-lagged model of openness and insomnia, the autoregressive paths of openness (*β* = 0.127, *p* < 0.01) and insomnia (*β* = 0.360, *p* < 0.001) were all significant. In this model, the cross-lagged path from openness at baseline to insomnia at follow-up (*β* = −0.053, *p* = 0.158) and the path from insomnia at baseline to openness at follow-up (*β* = −0.044, *p* = 0.337) was not significant. In the cross-lagged model of agreeableness and insomnia, the autoregressive paths of insomnia (*β* = 0.358, *p* < 0.001) was significant and the autoregressive paths of agreeableness (*β* = 0.030, *p* = 0.510) was not significant. In this model, the cross-lagged path from insomnia at baseline to agreeableness at follow-up (*β* = −0.122, *p* < 0.01) was significant and the path from agreeableness at baseline to insomnia at follow-up (*β* = −0.055, *p* = 0.135) was not significant. In the cross-lagged model of conscientiousness and insomnia, the autoregressive paths of conscientiousness (*β* = 0.107, *p* < 0.05) and insomnia (*β* = 0.341, *p* < 0.001) were all significant. In this model, the cross-lagged path from conscientiousness at baseline to insomnia at follow-up (*β* = −0.168, *p* < 0.001) was significant and the path from insomnia at baseline to conscientiousness at follow-up (*β* = −0.055, *p* = 0.318) was not significant. The results supported that personality traits, except for agreeableness and openness, had a statistically significant longitudinal association with insomnia at follow-up. In addition, insomnia at baseline had statistically significant longitudinal correlation with neuroticism, extraversion, and agreeableness at follow-up, as shown in Figure 2.

## Discussion

In this study, a prospective design was utilized to examine the bidirectional relationship between the five personality traits proposed by the Big Five theory and insomnia. Our findings are as follows. Neuroticism and extraversion had a bidirectional relationship with insomnia. Insomnia associated with agreeableness and conscientiousness in a one-way manner. The longitudinal correlations between openness and insomnia were not supported in this study.

For neuroticism, this research indicated that it had a bidirectional relationship with insomnia. As can be seen from the cross-lagged results, the effect of neuroticism on insomnia is stronger than that of insomnia on neuroticism. There are plenty of studies showing that neuroticism can affect insomnia (Vedaa et al., 2016; Dekker et al., 2017; Booker et al., 2018; Ren et al., 2019; Sørengaard et al., 2019; Holzinger et al., 2021; Rojo-Wissar et al., 2021; Zhang et al., 2021; Akram et al., 2023). People with high neuroticism tend to ruminate more, have more nightmares, be more prone to psychological rigidity, and be more likely to abuse caffeine and alcohol. All these factors can lead to more severe insomnia (Ren et al., 2019; Fabbri et al., 2022; Habibi Asgarabad et al., 2023; Ollila et al., 2024). And severe insomnia can lead to the occurrence of these problems. Severe insomnia is associated with psychological disorders and maladaptive behaviors in a bidirectional reinforcing manner, forming a vicious cycle involving multiple paths such as neurotransmitter imbalance, overactivity of the HPA axis, and impaired cognitive function (Baglioni et al., 2011; Buysse, 2013). Based on the characteristics of neuroticism, priority should be given to adjusting emotional problems for individuals with high neuroticism and insomnia, reducing the intervention of individual isolation, and conducting cognitive therapy for rumination behavior. From a clinical perspective, the bidirectional relationship between neuroticism and insomnia holds significant importance for intervention. Individuals with high neuroticism exacerbate insomnia symptoms through pathways such as rumination thinking, frequency of nightmares, psychological rigidity, and caffeine/alcohol abuse. Based on the special relationship between neuroticism and insomnia, the following intervention measures are available for those with high neuroticism and insomnia. For those with high rumination thinking modified CBT-I combined with imagery rehearsal therapy is adopted to deal with nightmares (Taylor et al., 2023; Delage et al., 2024). For those with overactive HPA axis, mindfulness stress reduction training is supplemented (Piber et al., 2022; Delage et al., 2024). For those who abuse alcohol and caffeine, priority is given to controlling their intake. For those with psychological rigidity, acceptance and commitment therapy is adopted (Habibi Asgarabad et al., 2023). For extraversion, this research also shows that it has a bidirectional relationship with insomnia. Unlike neuroticism, the cross-lagged results indicated that the effect of insomnia on extraversion was stronger than that of extraversion on insomnia. It’s often thought that extraverts get more social and emotional support, which is good for a good night’s sleep (Chung, 2017). Extraverts typically have positive, optimistic mood and high emotional stability. They are more likely to experience positive emotions such as happiness, excitement, and satisfaction and a positive emotional state can reduce stress and anxiety, promote physical and mental relaxation, and help reduce insomnia (Wang and Zeng, 2023). In addition, extraverts are generally more active and enjoy participating in a variety of activities and sports. Their active participation in physical and mental activities may help to burn energy, reduce stress, and insomnia (Goldberg et al., 2024). Some researchers have suggested that extraverts have a higher tendency to present hedonism and a lower tendency to ruminate about negative past events, both of which also reduce insomnia (Fabbri et al., 2022). Several studies have indicated that insomnia can impact emotional stability, and that extroverts are generally more prone to large mood swings which make them more susceptible (Li et al., 2024). Insomnia can impair an individual’s social competence, thereby impacting their interpersonal relationships and potentially inducing alterations in extraversion (Raman et al., 2024). From a clinical perspective, the bidirectional relationship between extraversion and insomnia holds significant implications for intervention. This research indicates that the negative impact of insomnia on extraversion is stronger than the protective effect of extraversion on insomnia. This suggests that clinicians should pay special attention to the impairment of social function in patients with insomnia. Extraverts usually obtain protection against insomnia through social support, optimistic emotions, and physical activities. However, insomnia may disrupt their emotional stability and social competence, forming a vicious cycle. In clinical practice, a dual-pronged strategy is needed: on one hand, emotional regulation training should be intensified for patients with extraverted insomnia; on the other hand, social participation should be maintained through group activities to prevent the deterioration of social function caused by insomnia.

For openness, this research reveals no significant longitudinal association between openness and insomnia. The present findings are in line with the outcomes of prior investigations (Holzinger et al., 2021; Mead et al., 2021; Rojo-Wissar et al., 2021). In general, openness means greater resilience, lower stress, and better mental and physical health (Ozer and Benet-Martínez, 2006). However, some of factors associated with openness (such as higher intelligence and study/work engagement) may be associated with insomnia. Higher intelligence is associated with insomnia, possibly because people with higher IQ show more rumination and cognitive activation (Geiger et al., 2010). These two distinct effects can explain why openness is not associated with insomnia. For the population under study in this research, they are likely to be greatly influenced by cultural and environmental factors. On one hand, in rural environments, due to the relatively closed information flow and traditional lifestyle, residents have limited opportunities to encounter new things and their educational levels are relatively low, which leads to the lack of obvious manifestations of openness (Atherton et al., 2024). On the other hand, the social structure and lifestyle of rural communities are usually relatively fixed, and there are fewer new things or cultural stimuli. Even if an individual has a high level of openness, the actual environment lacks the conditions to trigger exploratory behavior, thereby suppressing the potential impact of openness on psychological states. All these will affect the degree of association between openness and insomnia, thereby making it lose its statistical significance.

For agreeableness, this research manifests that it has a one-way relationship with insomnia. The cross-lagged findings indicated that baseline insomnia was negatively associated with follow-up agreeableness. The trait of agreeableness can be succinctly described as altruism, and empirical research has demonstrated that insomnia is associated with an emotional imbalance characterized by a decline in emotional empathy and impaired accuracy in identifying others’ emotions (Guadagni et al., 2018). These factors can exert a detrimental impact on agreeableness.

For conscientiousness, this research finds that it also has a one-way relationship with insomnia. The findings from cross-lagged analyses suggested that conscientiousness had a longitudinal negative effect on insomnia, whereas insomnia did not affect the changes of conscientiousness over time. The negative impact of conscientiousness on insomnia has been substantiated by numerous studies (Kim et al., 2015; Akram et al., 2019; Holzinger et al., 2021; Mead et al., 2021; Rojo-Wissar et al., 2021). It is commonly assumed that individuals with high levels of conscientiousness exhibit self-restraint and meticulousness in their daily activities, which can contribute to more positive subjective sleep experiences (Rojo-Wissar et al., 2021; Sørengaard et al., 2022). Moreover, individuals with higher conscientiousness tend to perceive their sleep in a more optimistic manner (Wang and Zeng, 2023). Furthermore, individuals exhibiting high levels of conscientiousness also demonstrate heightened orientations toward both future and past-positive experiences (Fabbri et al., 2022). All of these features lower the risk of insomnia. For individuals with low conscientiousness, behavioral training can be adopted to enhance their ability to structurally control daily activities, thereby reducing anxiety caused by procrastination and uncertainty (Carciofo, 2022; Fabbri et al., 2022).

In addition to personality traits, our findings also indicate a higher severity of insomnia among individuals with lower levels of education. Furthermore, it was observed that married individuals tend to experience a lower prevalence of insomnia compared to other demographic groups. These two findings are consistent with the results of previous studies (Zheng et al., 2018; Kawata et al., 2020; Alharbi et al., 2021). The researchers posit that the correlation may stem from married individuals exhibiting healthier lifestyles and better mental well-being (Kawata et al., 2020). However, the majority of participants in this study are older adults. In rural China, older unmarried individuals often accompanied with challenging economic circumstances, while widows and divorcees often suffer financial hardship. Less insomnia in the married population may be mediated through economic conditions. The impact of education level on insomnia is widely acknowledged, as it influences various factors such as income, access to healthcare, problem-solving abilities, and other related aspects (Kim et al., 2017).

In this study, the attrition process, including deaths, migration, and loss to follow-up, was meticulously recorded, and sensitivity analysis was conducted to assess the potential impact of missing data on the study’s research results. Although the analysis of this study indicated that the population lost to follow-up exhibited different characteristics, such as a lower average age and milder insomnia problems, these characteristics were consistent with the actual situation (the high mobility of young people led to a higher possibility of loss to follow-up, and the severity of insomnia was related to age). This might limit the applicability of the research results to a broader population, and loss to follow-up might reduce the statistical power of the analysis in this study. Additionally, this study conducted rigorous tests on the key statistical assumptions, such as normality, linearity, and multicollinearity, to ensure the validity and reliability of the research results. The normality of residuals was evaluated through graphical methods and statistical tests, and the results indicated that the residuals were approximately normally distributed. The linear relationship was tested through scatter plots and residual plots, revealing a slight U-shaped trend in some cross-lagged paths, though this did not significantly affect the overall model fit. However, the sample size (*N* = 482) of this study met the requirements of a large sample size for the cross-lagged model. Under the support of the central limit theorem, the asymptotic normality of the estimates accommodated weak non-linearity, ensuring the reliability of the parameter estimates. To thoroughly verify this, the Bootstrap method (1,000 samples) was used to calculate robust confidence intervals. The 95% confidence intervals of all key paths did not cross zero. Multicollinearity was evaluated through variance inflation factors (VIF), and all indicators were below the conventional threshold of 10. Since the model encompasses all possible time-lagged paths, it results in a saturated model with zero degrees of freedom. Despite some shortcomings, this study still holds significant value in multiple aspects. Firstly, the results of this study provide support for the growing body of evidence that insomnia among rural residents is longitudinally associated with some personality traits. Secondly, by documenting the process of loss to follow-up and rigorously testing statistical hypotheses, this study lays the foundation for further exploration of the underlying mechanisms of these associations in future research. If future studies can adopt larger sample sizes, improved follow-up protocols, and other analytical methods, they can further mitigate the impact of loss to follow-up on the research results and enhance the generalizability of the research findings in this field.

There are some limitations to this study. Firstly, reliance on self-reported data for personality and insomnia introduces potential biases, such as recall inaccuracies and social desirability effects. To address these limitations, future studies could integrate objective sleep assessments to validate self-reported insomnia metrics and incorporate informant reports to triangulate personality data. Secondly, this research was conducted only in rural areas of Shandong Province, China. It should be acknowledged that due to different cultural backgrounds, this will limit the universal applicability of our research results among urban residents and people from different countries and regions. If one wants to continue to explore this issue, future research should be conducted in multiple regions and involving multiple groups of people. Thirdly, there were uncontrolled variables that could potentially exert a significant impact on insomnia. Lastly, while this study provides evidence for the association between insomnia and personality, it does not fully explore the mechanisms underlying this relationship, particularly the role of mental health factors. Future research will aim to address these gaps and further investigate the mechanisms at play.

## Conclusion

To our knowledge, this study represents the first attempt to explore the longitudinal associations between personality traits and insomnia among rural Chinese residents using a prospective cohort design. This research contributes to the existing literature in the following ways. Firstly, it revealed a bidirectional relationship between neuroticism, extraversion, and insomnia. Specifically, individuals with high levels of neuroticism displayed more severe overall insomnia symptoms. The impact of neuroticism on insomnia was found to be stronger than the reverse effect. In the case of extraversion, those with low levels of this trait experienced more severe insomnia, and interestingly, the influence of insomnia on extraversion was more significant than that of extraversion on insomnia. Secondly, a one – way longitudinal association was identified between insomnia and agreeableness, as well as between conscientiousness and insomnia. Participants with severe insomnia showed lower levels of agreeableness, and those with low conscientiousness had more pronounced insomnia symptoms. However, the longitudinal correlations between openness and insomnia were not supported in this study. This study enhances our comprehension of the association between personality traits and insomnia, thereby facilitating further investigation into the causal link between insomnia and personality characteristics.

## Data Availability

The original contributions presented in the study are included in the article/supplementary material, further inquiries can be directed to the corresponding author.
